# 7-Benzyl-2,7-diaza­spiro­[4.4]nonan-1-one

**DOI:** 10.1107/S1600536811034301

**Published:** 2011-08-27

**Authors:** Huan-Mei Guo

**Affiliations:** a Microscale Science Institute, Weifang University, Weifang 261061, People’s Republic of China

## Abstract

In the title compound, C_14_H_18_N_2_O, both the spiro-linked five-membered rings adopt envelope conformations, with a C atom as the flap in one ring and an N atom in the other. The dihedral angle between the two four-atom planes is 80.46 (8)°. In the crystal, the mol­ecules are linked by N—H⋯O hydrogen bonds to generate *C*(4) chains propagating in [010].

## Related literature

For background to pyrrolidine derivatives, see: Kuroki *et al.* (1999 ▶); Hale *et al.* (2001 ▶); Shen *et al.* (2004 ▶).
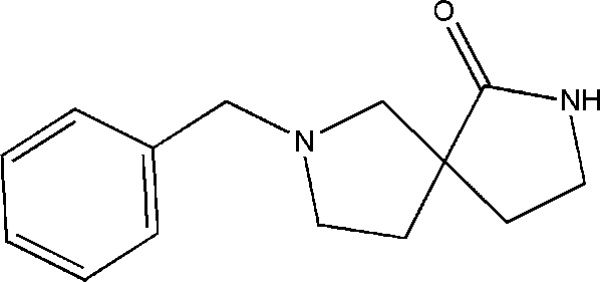


## Experimental

### 

#### Crystal data

C_14_H_18_N_2_O
*M* *_r_* = 230.30
Orthorhombic, 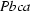

*a* = 9.630 (2) Å
*b* = 8.4322 (18) Å
*c* = 29.848 (7) Å
*V* = 2423.8 (9) Å^3^
*Z* = 8
Mo *K*α radiation
μ = 0.08 mm^−1^
*T* = 173 K
0.21 × 0.18 × 0.17 mm

#### Data collection

MM007-HF CCD (Saturn 724+) diffractometer
Absorption correction: multi-scan (*CrystalClear*; Rigaku, 2007 ▶) *T*_min_ = 0.983, *T*_max_ = 0.986
9156 measured reflections
2761 independent reflections
2495 reflections with *I* > 2σ(*I*)
*R*_int_ = 0.046

#### Refinement

*R*[*F* ^2^ > 2σ(*F* ^2^)] = 0.059
*wR*(*F* ^2^) = 0.120
*S* = 1.16
2761 reflections
154 parameters
H-atom parameters constrained
Δρ_max_ = 0.22 e Å^−3^
Δρ_min_ = −0.19 e Å^−3^

### 

Data collection: *CrystalClear* (Rigaku, 2007 ▶); cell refinement: *CrystalClear*; data reduction: *CrystalClear*; program(s) used to solve structure: *SHELXS97* (Sheldrick, 2008 ▶); program(s) used to refine structure: *SHELXL97* (Sheldrick, 2008 ▶); molecular graphics: *SHELXTL* (Sheldrick, 2008 ▶); software used to prepare material for publication: *SHELXTL*.

## Supplementary Material

Crystal structure: contains datablock(s) global, I. DOI: 10.1107/S1600536811034301/hb6338sup1.cif

Structure factors: contains datablock(s) I. DOI: 10.1107/S1600536811034301/hb6338Isup2.hkl

Supplementary material file. DOI: 10.1107/S1600536811034301/hb6338Isup3.cml

Additional supplementary materials:  crystallographic information; 3D view; checkCIF report

## Figures and Tables

**Table 1:** Hydrogen-bond geometry (Å, °)

*D*—H⋯*A*	*D*—H	H⋯*A*	*D*⋯*A*	*D*—H⋯*A*
N3—H3⋯O1^i^	0.88	2.14	2.9839 (19)	160

Symmetry code: (i) 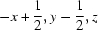.

**Fig. 1.**
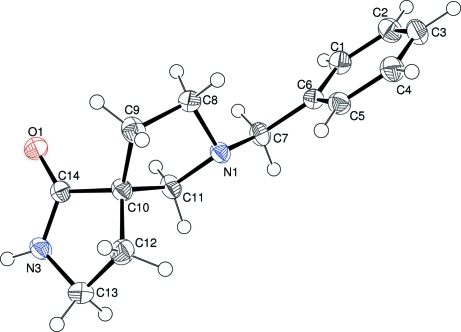
The molecular structure of (I) showing displacement ellipsoids at the 50% probability level.

## supplementary crystallographic information

### Comment

While a great number of pyrrolidines and their derivatives with specific substitution pattern are of particular interest, new methods for their preparation are needed; e.g. Kuroki *et al.*, (1999); Hale *et al.*, (2001); Shen *et al.*, (2004).  As part of our research work in this area, the title compound, (I), was synthesized, and herein we report the structure of it.

In the molecule (Fig. 1), all bond lengths and angles are within normal ranges. Atoms C8, C9, C10, and C11 lie in a plane (p1),with a maximum deviation of 0.01102 (11)Å for C10; atoms C10, C13, C14, and N3 lie in a plane (p2) too, the maximum deviation is 0.0045 (10)Å for N3. The dihedral angle between the two plans is 80.46 (8)°. The dihedral angles made by the phenyl ring with p1anes p1 and p2 are 53.56 (9)° and 50.21 (6)°, respectively. The structure exhibits intermolecular N3—H···O1 hydrogen bonding interactions (Table 1), which link the molecules into chains.

### Experimental

The title molecule, C_14_H_18_N_2_O_1_, was synthesized from methyl 1-benzyl-3-(cyanomethyl) pyrrolidine-3-carboxylate and Raney Ni (*w*/*w* = 4: 1) in methanol under H_2_ (50 Psi) atmosphere at room temperature.  Colourless blocks of (I) were obtained by recrystallization from ethanol at room temperature.

### Refinement

All H atoms were fixed geometrically and allowed to ride on their attached atoms, the C—H distances is in the range 0.95–0.98 Å, and with *U*_iso_(H) = 1.2*U*_eq_(C); the N—H distances is 0.88 Å, with *U*_iso_(H) = 1.2*U*_eq_(N).

### Crystal data

**Table:**

C_14_H_18_N_2_O	*F*(000) = 992
*M**_r_* = 230.30	*D*_x_ = 1.262 Mg m^−^^3^
Orthorhombic, *P**b**c**a*	Mo *K*α radiation, λ = 0.71073 Å
Hall symbol: -P 2ac 2ab	Cell parameters from 6994 reflections
*a* = 9.630 (2) Å	θ = 1.4–27.5°
*b* = 8.4322 (18) Å	µ = 0.08 mm^−^^1^
*c* = 29.848 (7) Å	*T* = 173 K
*V* = 2423.8 (9) Å^3^	Block, colorless
*Z* = 8	0.21 × 0.18 × 0.17 mm

### Data collection

**Table:**

MM007-HF CCD (Saturn 724+) diffractometer	2761 independent reflections
Radiation source: rotating anode	2495 reflections with *I* > 2σ(*I*)
Confocal	*R*_int_ = 0.046
ω scans at fixed χ = 45°	θ_max_ = 27.5°, θ_min_ = 2.5°
Absorption correction: multi-scan (*CrystalClear*; Rigaku, 2007)	*h* = −7→12
*T*_min_ = 0.983, *T*_max_ = 0.986	*k* = −7→10
9156 measured reflections	*l* = −38→38

### Refinement

**Table:**

Refinement on *F*^2^	Primary atom site location: structure-invariant direct methods
Least-squares matrix: full	Secondary atom site location: difference Fourier map
*R*[*F*^2^ > 2σ(*F*^2^)] = 0.059	Hydrogen site location: inferred from neighbouring sites
*wR*(*F*^2^) = 0.120	H-atom parameters constrained
*S* = 1.16	*w* = 1/[σ^2^(*F*_o_^2^) + (0.030*P*)^2^ + 1.3429*P*] where *P* = (*F*_o_^2^ + 2*F*_c_^2^)/3
2761 reflections	(Δ/σ)_max_ < 0.001
154 parameters	Δρ_max_ = 0.22 e Å^−^^3^
0 restraints	Δρ_min_ = −0.19 e Å^−^^3^

### Special details

**Table:**

Geometry. All e.s.d.'s (except the e.s.d. in the dihedral angle between two l.s. planes) are estimated using the full covariance matrix. The cell e.s.d.'s are taken into account individually in the estimation of e.s.d.'s in distances, angles and torsion angles; correlations between e.s.d.'s in cell parameters are only used when they are defined by crystal symmetry. An approximate (isotropic) treatment of cell e.s.d.'s is used for estimating e.s.d.'s involving l.s. planes.
Refinement. Refinement of *F*^2^ against ALL reflections. The weighted *R*-factor *wR* and goodness of fit *S* are based on *F*^2^, conventional *R*-factors *R* are based on *F*, with *F* set to zero for negative *F*^2^. The threshold expression of *F*^2^ > σ(*F*^2^) is used only for calculating *R*-factors(gt) *etc*. and is not relevant to the choice of reflections for refinement. *R*-factors based on *F*^2^ are statistically about twice as large as those based on *F*, and *R*- factors based on ALL data will be even larger.

### Fractional atomic coordinates and isotropic or equivalent isotropic displacement parameters (Å^2^)

**Table:**

	*x*	*y*	*z*	*U*_iso_*/*U*_eq_	
O1	0.27133 (12)	0.12904 (14)	0.47500 (4)	0.0295 (3)	
N1	0.48470 (14)	0.35120 (16)	0.37041 (4)	0.0226 (3)	
N3	0.39956 (15)	−0.08606 (16)	0.45320 (5)	0.0265 (3)	
H3	0.3485	−0.1589	0.4664	0.032*	
C1	0.39718 (18)	0.6967 (2)	0.29710 (6)	0.0279 (4)	
H1	0.3010	0.6777	0.2924	0.033*	
C2	0.4556 (2)	0.8372 (2)	0.28234 (6)	0.0324 (4)	
H2	0.3996	0.9135	0.2675	0.039*	
C3	0.5953 (2)	0.8667 (2)	0.28919 (6)	0.0319 (4)	
H3A	0.6352	0.9636	0.2793	0.038*	
C4	0.67658 (19)	0.7545 (2)	0.31053 (6)	0.0305 (4)	
H4	0.7726	0.7741	0.3153	0.037*	
C5	0.61789 (17)	0.61353 (19)	0.32491 (6)	0.0255 (4)	
H5	0.6746	0.5365	0.3392	0.031*	
C6	0.47727 (17)	0.58284 (19)	0.31871 (5)	0.0228 (3)	
C7	0.41177 (18)	0.4289 (2)	0.33361 (6)	0.0259 (4)	
H7B	0.3149	0.4501	0.3430	0.031*	
H7A	0.4085	0.3554	0.3078	0.031*	
C8	0.48226 (19)	0.4420 (2)	0.41228 (5)	0.0266 (4)	
H8A	0.3889	0.4870	0.4178	0.032*	
H8B	0.5508	0.5294	0.4115	0.032*	
C9	0.5201 (2)	0.3210 (2)	0.44804 (6)	0.0315 (4)	
H9A	0.4680	0.3417	0.4760	0.038*	
H9B	0.6207	0.3248	0.4547	0.038*	
C10	0.47966 (17)	0.15873 (19)	0.42824 (5)	0.0237 (4)	
C11	0.41627 (19)	0.2024 (2)	0.38238 (5)	0.0264 (4)	
H11B	0.4358	0.1191	0.3599	0.032*	
H11A	0.3145	0.2169	0.3848	0.032*	
C12	0.59701 (18)	0.0370 (2)	0.42444 (6)	0.0310 (4)	
H12A	0.6471	0.0487	0.3957	0.037*	
H12B	0.6640	0.0489	0.4494	0.037*	
C13	0.52313 (19)	−0.1232 (2)	0.42691 (6)	0.0317 (4)	
H13A	0.4981	−0.1622	0.3967	0.038*	
H13B	0.5814	−0.2036	0.4421	0.038*	
C14	0.37117 (16)	0.06900 (19)	0.45541 (5)	0.0220 (3)	

### Atomic displacement parameters (Å^2^)

**Table:**

	*U*^11^	*U*^22^	*U*^33^	*U*^12^	*U*^13^	*U*^23^
O1	0.0269 (6)	0.0299 (7)	0.0317 (6)	0.0042 (5)	0.0045 (5)	0.0012 (5)
N1	0.0255 (7)	0.0193 (7)	0.0231 (7)	−0.0027 (6)	−0.0004 (5)	0.0010 (5)
N3	0.0277 (7)	0.0197 (7)	0.0321 (8)	−0.0019 (6)	0.0035 (6)	0.0033 (6)
C1	0.0259 (8)	0.0299 (9)	0.0279 (8)	0.0013 (8)	−0.0008 (7)	0.0021 (7)
C2	0.0361 (10)	0.0283 (9)	0.0329 (9)	0.0047 (8)	−0.0023 (8)	0.0071 (7)
C3	0.0383 (10)	0.0255 (9)	0.0320 (9)	−0.0055 (8)	0.0031 (8)	0.0054 (7)
C4	0.0273 (9)	0.0283 (9)	0.0358 (9)	−0.0047 (7)	0.0024 (7)	−0.0001 (7)
C5	0.0246 (8)	0.0232 (8)	0.0289 (9)	0.0013 (7)	0.0008 (7)	0.0015 (7)
C6	0.0245 (8)	0.0214 (8)	0.0226 (8)	−0.0004 (7)	0.0007 (6)	−0.0006 (6)
C7	0.0244 (8)	0.0258 (9)	0.0276 (8)	−0.0035 (7)	−0.0021 (6)	0.0036 (7)
C8	0.0308 (9)	0.0227 (8)	0.0263 (8)	−0.0014 (7)	0.0028 (7)	−0.0020 (7)
C9	0.0399 (10)	0.0272 (9)	0.0273 (9)	−0.0090 (8)	−0.0052 (7)	0.0006 (7)
C10	0.0265 (8)	0.0209 (8)	0.0237 (8)	−0.0025 (7)	−0.0016 (6)	0.0012 (6)
C11	0.0307 (9)	0.0240 (8)	0.0245 (8)	−0.0058 (7)	−0.0035 (7)	0.0032 (7)
C12	0.0264 (9)	0.0344 (10)	0.0324 (9)	0.0023 (8)	0.0053 (7)	0.0032 (8)
C13	0.0352 (10)	0.0260 (9)	0.0341 (9)	0.0066 (8)	0.0034 (8)	−0.0010 (7)
C14	0.0218 (8)	0.0228 (8)	0.0214 (7)	−0.0004 (7)	−0.0019 (6)	0.0010 (6)

### Geometric parameters (Å, °)

**Table:**

O1—C14	1.2341 (19)	C7—H7B	0.9900
N1—C7	1.459 (2)	C7—H7A	0.9900
N1—C11	1.462 (2)	C8—C9	1.521 (2)
N1—C8	1.466 (2)	C8—H8A	0.9900
N3—C14	1.337 (2)	C8—H8B	0.9900
N3—C13	1.460 (2)	C9—C10	1.540 (2)
N3—H3	0.8800	C9—H9A	0.9900
C1—C2	1.383 (2)	C9—H9B	0.9900
C1—C6	1.390 (2)	C10—C14	1.524 (2)
C1—H1	0.9500	C10—C12	1.531 (2)
C2—C3	1.384 (3)	C10—C11	1.544 (2)
C2—H2	0.9500	C11—H11B	0.9900
C3—C4	1.384 (3)	C11—H11A	0.9900
C3—H3A	0.9500	C12—C13	1.529 (2)
C4—C5	1.384 (2)	C12—H12A	0.9900
C4—H4	0.9500	C12—H12B	0.9900
C5—C6	1.391 (2)	C13—H13A	0.9900
C5—H5	0.9500	C13—H13B	0.9900
C6—C7	1.510 (2)		
			
C7—N1—C11	110.62 (13)	H8A—C8—H8B	108.9
C7—N1—C8	113.55 (13)	C8—C9—C10	105.45 (14)
C11—N1—C8	103.47 (13)	C8—C9—H9A	110.7
C14—N3—C13	113.77 (14)	C10—C9—H9A	110.7
C14—N3—H3	123.1	C8—C9—H9B	110.7
C13—N3—H3	123.1	C10—C9—H9B	110.7
C2—C1—C6	120.90 (16)	H9A—C9—H9B	108.8
C2—C1—H1	119.5	C14—C10—C12	102.28 (13)
C6—C1—H1	119.5	C14—C10—C9	114.22 (14)
C1—C2—C3	120.16 (17)	C12—C10—C9	115.95 (14)
C1—C2—H2	119.9	C14—C10—C11	108.61 (13)
C3—C2—H2	119.9	C12—C10—C11	112.71 (14)
C4—C3—C2	119.67 (17)	C9—C10—C11	103.19 (13)
C4—C3—H3A	120.2	N1—C11—C10	104.07 (13)
C2—C3—H3A	120.2	N1—C11—H11B	110.9
C3—C4—C5	119.94 (17)	C10—C11—H11B	110.9
C3—C4—H4	120.0	N1—C11—H11A	110.9
C5—C4—H4	120.0	C10—C11—H11A	110.9
C4—C5—C6	121.06 (16)	H11B—C11—H11A	109.0
C4—C5—H5	119.5	C13—C12—C10	104.21 (14)
C6—C5—H5	119.5	C13—C12—H12A	110.9
C1—C6—C5	118.26 (15)	C10—C12—H12A	110.9
C1—C6—C7	119.90 (15)	C13—C12—H12B	110.9
C5—C6—C7	121.82 (15)	C10—C12—H12B	110.9
N1—C7—C6	113.99 (13)	H12A—C12—H12B	108.9
N1—C7—H7B	108.8	N3—C13—C12	102.45 (13)
C6—C7—H7B	108.8	N3—C13—H13A	111.3
N1—C7—H7A	108.8	C12—C13—H13A	111.3
C6—C7—H7A	108.8	N3—C13—H13B	111.3
H7B—C7—H7A	107.7	C12—C13—H13B	111.3
N1—C8—C9	104.13 (14)	H13A—C13—H13B	109.2
N1—C8—H8A	110.9	O1—C14—N3	125.71 (15)
C9—C8—H8A	110.9	O1—C14—C10	125.64 (15)
N1—C8—H8B	110.9	N3—C14—C10	108.61 (14)
C9—C8—H8B	110.9		
			
C6—C1—C2—C3	−0.4 (3)	C7—N1—C11—C10	−165.66 (13)
C1—C2—C3—C4	0.6 (3)	C8—N1—C11—C10	−43.71 (16)
C2—C3—C4—C5	0.0 (3)	C14—C10—C11—N1	149.00 (13)
C3—C4—C5—C6	−0.7 (3)	C12—C10—C11—N1	−98.41 (16)
C2—C1—C6—C5	−0.3 (2)	C9—C10—C11—N1	27.42 (17)
C2—C1—C6—C7	−178.67 (16)	C14—C10—C12—C13	28.06 (17)
C4—C5—C6—C1	0.9 (2)	C9—C10—C12—C13	153.01 (15)
C4—C5—C6—C7	179.16 (16)	C11—C10—C12—C13	−88.38 (16)
C11—N1—C7—C6	−179.16 (13)	C14—N3—C13—C12	17.26 (19)
C8—N1—C7—C6	65.02 (18)	C10—C12—C13—N3	−27.63 (17)
C1—C6—C7—N1	−156.04 (15)	C13—N3—C14—O1	178.80 (16)
C5—C6—C7—N1	25.7 (2)	C13—N3—C14—C10	0.86 (19)
C7—N1—C8—C9	162.37 (14)	C12—C10—C14—O1	163.51 (16)
C11—N1—C8—C9	42.41 (16)	C9—C10—C14—O1	37.4 (2)
N1—C8—C9—C10	−24.29 (18)	C11—C10—C14—O1	−77.1 (2)
C8—C9—C10—C14	−119.53 (15)	C12—C10—C14—N3	−18.55 (17)
C8—C9—C10—C12	121.90 (16)	C9—C10—C14—N3	−144.63 (14)
C8—C9—C10—C11	−1.82 (18)	C11—C10—C14—N3	100.81 (15)

### Hydrogen-bond geometry (Å, °)

**Table:**

*D*—H···*A*	*D*—H	H···*A*	*D*···*A*	*D*—H···*A*
N3—H3···O1^i^	0.88	2.14	2.9839 (19)	160

Symmetry codes: (i) −*x*+1/2, *y*−1/2, *z*.
